# Complete plastid genomes from *Ophioglossum californicum*, *Psilotum nudum*, and *Equisetum hyemale* reveal an ancestral land plant genome structure and resolve the position of Equisetales among monilophytes

**DOI:** 10.1186/1471-2148-13-8

**Published:** 2013-01-11

**Authors:** Felix Grewe, Wenhu Guo, Emily A Gubbels, A Katie Hansen, Jeffrey P Mower

**Affiliations:** 1 Center for Plant Science Innovation, University of Nebraska, Lincoln, NE, USA; 2 Department of Agronomy and Horticulture, University of Nebraska, Lincoln, NE, USA; 3 School of Biological Sciences, University of Nebraska, Lincoln, NE, USA; 4 Present address: College of Natural Sciences, The University of Texas at Austin, Austin, TX, USA

## Abstract

**Background:**

Plastid genome structure and content is remarkably conserved in land plants. This widespread conservation has facilitated taxon-rich phylogenetic analyses that have resolved organismal relationships among many land plant groups. However, the relationships among major fern lineages, especially the placement of Equisetales, remain enigmatic.

**Results:**

In order to understand the evolution of plastid genomes and to establish phylogenetic relationships among ferns, we sequenced the plastid genomes from three early diverging species: *Equisetum hyemale* (Equisetales), *Ophioglossum californicum* (Ophioglossales), and *Psilotum nudum* (Psilotales). A comparison of fern plastid genomes showed that some lineages have retained inverted repeat (IR) boundaries originating from the common ancestor of land plants, while other lineages have experienced multiple IR changes including expansions and inversions. Genome content has remained stable throughout ferns, except for a few lineage-specific losses of genes and introns. Notably, the losses of the *rps16* gene and the rps12i346 intron are shared among Psilotales, Ophioglossales, and Equisetales, while the gain of a mitochondrial *atp1* intron is shared between Marattiales and Polypodiopsida. These genomic structural changes support the placement of Equisetales as sister to Ophioglossales + Psilotales and Marattiales as sister to Polypodiopsida. This result is augmented by some molecular phylogenetic analyses that recover the same relationships, whereas others suggest a relationship between Equisetales and Polypodiopsida.

**Conclusions:**

Although molecular analyses were inconsistent with respect to the position of Marattiales and Equisetales, several genomic structural changes have for the first time provided a clear placement of these lineages within the ferns. These results further demonstrate the power of using rare genomic structural changes in cases where molecular data fail to provide strong phylogenetic resolution.

## Background

The plastid genome has remained remarkably conserved throughout the evolution of land plants (reviewed in [1-3]). Genomes from diverse land plant lineages—including seed plants, ferns, lycophytes, hornworts, mosses, and liverworts—have a similar repertoire of genes that generally encode for proteins involved in photosynthesis or gene expression. The order of these plastid genes has remained consistent for most species, such that large syntenic tracks can be easily identified between genomes. Furthermore, most plastid genomes have a quadripartite structure involving a large single-copy (LSC) and a small single-copy (SSC) region separated by two copies of an inverted repeat (IR). Although these generalities apply to most land plants, exceptions certainly exist, such as the convergent loss of photosynthetic genes from parasitic plants [4-6] or *ndh* genes from several lineages [7,8], the highly rearranged genomes of some species [9-11], and the independent loss of one copy of the IR in several groups [8,11-13].

Because of the conserved structure and content of plastid genomes, its sequences have been favored targets for many plant phylogenetic analyses (e.g., [14,15]). Through extensive sequencing from phylogenetically diverse species, our understanding of the relationships between the major groups of land plants has greatly improved in recent years [15-19]. However, there are a few nodes whose position remains elusive, most notably that of the Gnetales [7,20] and the horsetails [16,18,21]. Horsetails (Equisetopsida) are particularly enigmatic because until recently [21] their morphology had been considered to be ‘primitive’ among vascular plants, and consequently they were grouped with the “fern allies” rather than with the “true” ferns. Recent molecular and morphological evidence now unequivocally support the inclusion of horsetails in ferns *sensu lato* (Monilophyta or Moniliformopses), which also encompasses whisk ferns and ophioglossoid ferns (Psilotopsida), marattioid ferns (Marattiopsida), and leptosporangiate ferns (Polypodiopsida) [16,18,21].

Despite this progress, the relationships among fern groups, especially horsetails, have been difficult to resolve with confidence. Many molecular phylogenetic analyses have suggested that horsetails are sister to marattioid ferns [16,21-23], while other analyses using different data sets and/or optimality criteria have suggested a position either with leptosporangiate ferns, with *Psilotum*, or as the sister group to all living monilophytes [3,18,21,24,25]. However, these various analyses rarely place *Equisetum* with strong statistical support. This phylogenetic uncertainty stems from at least two main issues. First, Equisetopsida is an ancient lineage dating back more than 300 million years, but extant (crown group) members are limited to *Equisetum*, which diversified only within the last 60 million years [26]. Second, substitution rates in the plastid (and mitochondrial) genome appear to be elevated in horsetails compared with other early diverging ferns (note the long branches in [21,22,25,27]). Consequently, molecular phylogenetic analyses produce a long evolutionary branch leading to *Equisetum*, a problem that can lead to long-branch attraction artifacts (reviewed in [28]).

In cases where molecular phylogenetic results are inconsistent, the use of rare genomic structural changes, such as large-scale inversions and the presence or absence of genes and introns, can provide independent indications of organismal relationships [29]. One notable example used the differential distribution of three mitochondrial introns to infer that liverworts were the earliest diverging land plant lineage [30]. Other studies have identified diagnostic inversions in the plastid genomes of euphyllophytes [31] and monilophytes [18]. Unfortunately, complete plastid genomes are currently lacking from several important fern clades, preventing a comprehensive study of the utility of plastid structural changes in resolving fern relationships.

In this study, we sequenced three additional fern plastid genomes: the ophioglossoid fern *Ophioglossum californicum*, the horsetail *Equisetum hyemale*, and the whisk fern *Psilotum nudum*. By sequencing the first ophioglossoid fern and a second horsetail (*E. hyemale* belongs to a different subgenus than the previously sequenced *E. arvense*[26,32]), we expected that this increased sampling would allow us to evaluate diversity in plastid genome structure and content and to resolve fern relationships using sequence and structural characters.

## Results and discussion

### Static vs. dynamic plastome structural evolution in monilophytes

**Figure 1:**
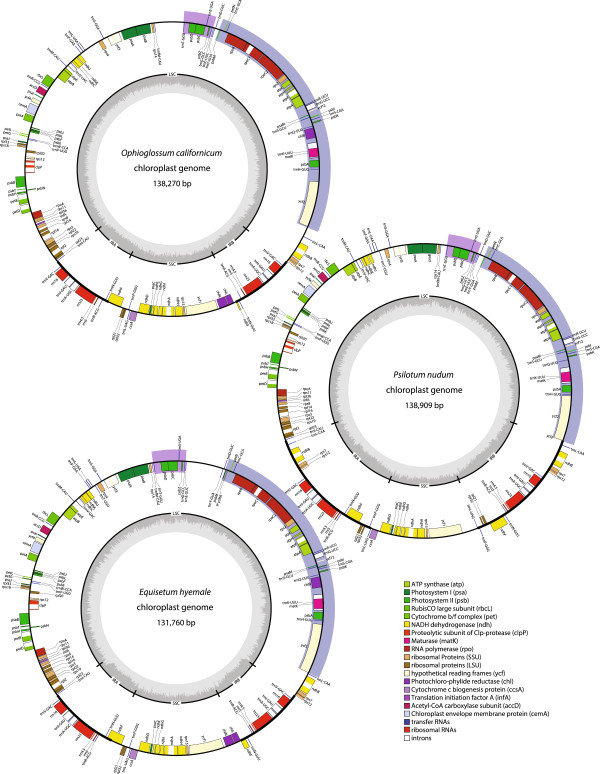
**Plastome maps for newly sequenced monilophytes.** Boxes on the inside and outside of the outer circle represent genes transcribed clockwise and anti-clockwise, respectively. The inner circle displays the GC content represented by dark gray bars. The location of the IRs are marked on the inner circle and represented by a thicker black line in the outer circle. The large euphyllophyte LSC inversion and the small monilophyte LSC inversion are highlighted on the outer circle by blue and purple bars, respectively.

The three chloroplast DNA (cpDNA) sequences from *Ophioglossum californicum*, *Psilotum nudum,* and *Equisetum hyemale* (Figure 1) have a typical circularly mapping structure containing the LSC and SSC separated by two IRs. All three genomes contain the large LSC inversion (from *psbM* to *ycf2*) found in euphyllophytes as well as the smaller LSC inversion (from *trnG*-GCC to *trnT*-GGU) that is specific to monilophytes (Figure 1; [18,31]).

We compared the general structural features of these three new genomes to other available monilophyte and lycophyte cpDNAs (Table 1). The 131,760 bp *E. hyemale* genome is the smallest sequenced to date, closest in size to that from *E. arvense* (133,309 bp). The *O. californicum* and *P. nudum* genomes are slightly larger, at 138,270 bp and 138,909 bp, respectively, whereas all other published monilophytes are >150 kb. The reduced genome sizes in *Equisetum, Ophioglossum,* and *Psilotum* are due to smaller SSCs and IRs compared to other species. Despite the similar genome sizes between *O. californicum* and *P. nudum*, the IR and SSC sizes in *O. californicum* are more similar to *Equisetum* than to *P. nudum*. GC content is quite variable among monilophytes, ranging from 33% in *E. arvense* to 42% in *Ophioglossum* and *Angiopteris* (although the unlisted polypod *Cheilanthes lindheimeri* has 43% GC).

**Table 1:** General features of cpDNA from selected lycophytes and monilophytes

	**Lycopodiophyta**		**Psilotopsida**		**Equisetopsida**		**Marattiopsida**	**Polypodiopsida**		
	***Isoetes***	***Huperzia***	***Ophioglossum***	***Psilotum***	***Equisetum***	***Equisetum***	***Angiopteris***	***Alsophila***	***Adiantum***	***Pteridium***
	***flaccida***	***lucidula***	***californicum***	***nudum***	***hyemale***	***arvense***	***evecta***	***spinulosa***	***capillus-veneris***	***aquilinum***
Accession	GU191333	AY660566	KC117178	KC117179	KC117177	GU191334	DQ821119	FJ556581	AY178864	HM535629
Size (bp)	145303	154373	138270	138909	131760	133309	153901	156661	150568	152362
LSC (bp)	91862	104088	99058	84674	92580	93542	89709	86308	82282	84335
SSC (bp)	27205	19657	19662	16329	18994	19469	22086	21623	21392	21259
IRs (bp)	13118	15314	9775	18953	10093	10149	21053	24365	23447	23384
G/C (%)	37.9	36.3	42.2	36.0	33.7	33.4	35.5	40.4	42.0	41.5
Genes	118	121	120	118	121	121	122	117	116	116
tRNAs	32	31	32	33	33	33	33	28	28	28
rRNAs	4	4	4	4	4	4	4	4	4	4
Protein coding	82	86	84	81	84	84	85	85	84	84
Introns	21	22	19	19	17	18	22	20	20	20

**Figure 2:**
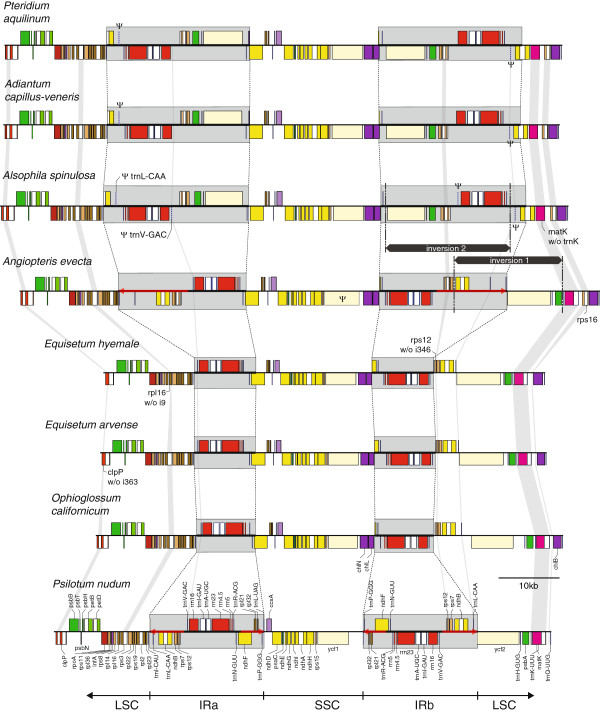
**Comparison of the IR and adjacent sequences from monilophytes.** A section of the plastid genome from *clpP* to *trnQ*-UUG is presented for selected monilophytes. The section includes the IR, SSC, and parts of the LSC. Genes shown above or below the lines indicate direction of transcription to the right or the left, respectively. The IR is marked by gray boxes, inferred IR extensions are shown by red arrows, and inferred inversions leading to the specific gene arrangement in Polypodiopsida are denoted by black bars. Molecular apomorphies based on gene and intron losses are highlighted by vertical gray lines. Maps are drawn approximately to scale. Color coding of genes corresponds to the legend shown in Figure 1.

**Figure 3:**
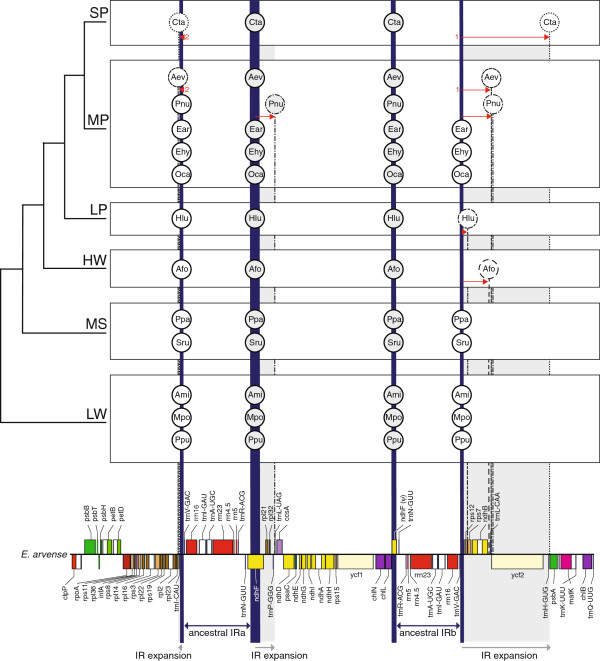
**Evolution of inverted repeat borders in selected land plants.** Species names are abbreviated in circles. Vertical lines depict the borders of the IR relative to the detailed gene map from *E. arvense* shown at bottom. Thick, solid vertical lines in dark blue mark the putative ancestral IR borders. Thin, dashed vertical lines and circles indicate the IR borders in species that deviate from the ancestral position. Horizontal arrows indicate the extent and direction of IR expansion. Numbers at the arrow tails define the order of successive expansions. All non-seed plant cpDNAs were included, except for *Isoetes*, *Selaginella*, and Polypodiopsida because their genomes have gene order rearrangements that make an alignment impossible. Included species: *Cycas taitungensis* (Cta), *Angiopteris evecta* (Aev), *Psilotum nudum* (Pnu), *Equisetum arvense* (Ear), *Equisetum hyemale* (Ehy), *Ophioglossum californicum* (Oca), *Huperzia lucidula* (Hlu), *Anthoceros formosae* (Afo), *Physcomitrella patens* (Ppa), *Syntrichia ruralis* (Sru), *Aneura mirabilis* (Ami), *Marchantia polymorpha* (Mpo), *Ptilidium pulcherrimum* (Ppu). Higher group names: seed plants (SP), monilophytes (MP), lycophytes (LP), hornworts (HW), mosses (MS), liverworts (LW).

A close inspection of the IRs among the five major groups of monilophytes (Psilotales, Ophioglossales, Equisetales, Marattiales and Polypodiopsida) reveals a dichotomous evolutionary history involving boundary shifts and inversions in some lineages and stasis in other lineages (Figures 2 and 3). The IRs in *Ophioglossum* and in both *Equisetum* plastomes contain the same complement of genes encoding all four plastid rRNAs and five tRNAs. The IR boundaries are also similar among these three species, placing *trnN*-GUU adjacent to either *ndhF* or *chlL* at the IR/SSC borders and *trnV*-GAC next to either *trnI*-CAU or the 3′-half of *rps12* at the IR/LSC borders. The exact border breakpoints differ slightly in each genome but generally terminate within the *ndhF* and/or *chlL* genes, creating a second fragmented copy of these genes. Interestingly, the gene adjacencies at the IR borders in *Ophioglossum* and *Equisetum* are virtually identical to those found outside the monilophytes, including the lycophyte *Huperzia lucidula*, the mosses *Physcomitrella patens* and *Syntrichia ruralis*, and the liverworts *Aneura mirabilis, Marchantia polymorpha,* and *Ptilidium pulcherrimum* (Figure 3). The similar IR borders among diverse vascular and non-vascular plants can be most parsimoniously explained by the plesiomorphic retention of this arrangement inherited from the land plant common ancestor.

In contrast to the static arrangement discussed above, the IRs among *Psilotum, Angiopteris*, and Polypodiopsida are more variable (Figures 2 and 3). The 19 kb IR in *P. nudum* includes nine additional genes due to expansion into one end of the SSC (gaining *ndhF*, *rpl21*, *rpl32*, *trnP*-GGG, and *trnL-*UAG) and into one end of the LSC (gaining *rps12*, *rps7*, *ndhB*, and *trnL*-CAA). The *A. evecta* IR exhibits intermediate characteristics: the IR/SSC border has retained the general ancestral position after *trnN*-GUU, but the IR has expanded twice into the LSC, adding *rps12*, *rps7*, *ndhB*, and *trnL*-CAA from one end of the LSC (similar to *Psilotum*) and *trnI*-CAU from the other end (unique to *A. evecta*). IRs among Polypodiopsida are more complex in origin, involving at least three major changes relative to the vascular plant ancestor. The unique gene orders within the IR and LSC can be most easily explained by an expansion of the IR to *trnL*-CAA (similar to *Psilotum* and *Angiopteris*), followed by two overlapping inversions (Figure 2; [33]). The first inversion appears to have involved a section from *ndhB* in the IR to *psbA* in the LSC. The second inversion spanned *trnR-*ACG through the inverted *ycf2* gene, which also included the previously inverted *psbA* and *trnH*-GUG genes but not the inverted pseudo-*trnL*-CAA or *ndhB* genes.

### Limited gene and intron content variation among monilophytes

A comparison of gene and intron content among representative monilophye and lycophyte plastomes indicates a conservative evolutionary history involving no gains and few losses (Tables 1 and 2). Some of the differences in total gene and intron numbers among species are due to differential duplication of a few genes after IR expansion in several lineages (Figure 2). Counting duplicated genes only once, the number of plastid-encoded genes varies from 116 to 122 due to minor changes in the set of tRNAs or protein-coding genes, while the number of introns ranges from 17 to 22 (Table 1).

**Table 2:** **Comparison of gene and intron content of cpDNAs from selected lycophytes and monilophytes**^**(a)**^

	**Gene/intron**	**If**	**Hl**	**Oc**	**Pn**	**Eh**	**Ea**	**Ae**	**As**	**Ac**	**Pa**		**Gene/intron**	**If**	**Hl**	**Oc**	**Pn**	**Eh**	**Ea**	**Ae**	**As**	**Ac**	**Pa**
**Transfer**	trnA-UGC	**+**	**+**	**+**	**+**	**+**	**+**	**+**	**+**	**+**	**+**	**ATP**	atpA	**+**	**+**	**+**	**+**	**+**	**+**	**+**	**+**	**+**	**+**
**RNAs**	**trnAUGCi38**	**+**	**+**	**+**	**+**	**+**	**+**	**+**	**+**	**+**	**+**	**synthase**	atpB	**+**	**+**	**+**	**+**	**+**	**+**	**+**	**+**	**+**	**+**
	trnC-GCA	**+**	**+**	**+**	**+**	**+**	**+**	**+**	**+**	**+**	**+**		atpE	**+**	**+**	**+**	**+**	**+**	**+**	**+**	**+**	**+**	**+**
	trnD-GUC	**+**	**+**	**+**	**+**	**+**	**+**	**+**	**+**	**+**	**+**		atpF	**+**	**+**	**+**	**+**	**+**	**+**	**+**	**+**	**+**	**+**
	trnE-UUC	**+**	**+**	**+**	**+**	**+**	**+**	**+**	**+**	**+**	**+**		**atpFi145**	**+**	**+**	**+**	**+**	**+**	**+**	**+**	**+**	**+**	**+**
	trnF-GAA	**+**	**+**	**+**	**+**	**+**	**+**	**+**	**+**	**+**	**+**		atpH	**+**	**+**	**+**	**+**	**+**	**+**	**+**	**+**	**+**	**+**
	trnfM-CAU	**+**	**+**	**+**	**+**	**+**	**+**	**+**	**+**	**+**	**+**		atpI	**+**	**+**	**+**	**+**	**+**	**+**	**+**	**+**	**+**	**+**
	trnG-GCC	**+**	**+**	**+**	**+**	**+**	**+**	**+**	**+**	**+**	**+**	**Chlorophyll**	chlB	**+**	**+**	**+**	**-**	**+**	**+**	**+**	**+**	**+**	**+**
	trnG-UCC	**+**	**+**	**+**	**+**	**+**	**+**	**+**	**+**	**+**	**+**	**biosynthesis**	chlL	**+**	**+**	**+**	**-**	**+**	**+**	**+**	**+**	**+**	**+**
	**trnGUCCi23**	**+**	**+**	**+**	**+**	**+**	**+**	**+**	**+**	**+**	**+**		chlN	**+**	**+**	**+**	**-**	**+**	**+**	**+**	**+**	**+**	**+**
	trnH-GUG	**+**	**+**	**+**	**+**	**+**	**+**	**+**	**+**	**+**	**+**	**NADH**	ndhA	**+**	**+**	**+**	**+**	**+**	**+**	**+**	**+**	**+**	**+**
	trnI-CAU	**+**	**+**	**+**	**+**	**+**	**+**	**+**	**+**	**+**	**+**	**dehydrogenase**	**ndhAi556**	**+**	**+**	**+**	**+**	**+**	**+**	**+**	**+**	**+**	**+**
	trnI-GAU	**+**	**+**	**+**	**+**	**+**	**+**	**+**	**+**	**+**	**+**		ndhB	**+**	**+**	**+**	**+**	**+**	**+**	**+**	**+**	**+**	**+**
	**trnIGAUi37**	**+**	**+**	**+**	**+**	**+**	**+**	**+**	**+**	**+**	**+**		**ndhBi726**	**+**	**+**	**+**	**+**	**+**	**+**	**+**	**+**	**+**	**+**
	trnK-UUU	**+**	**+**	**+**	**+**	**+**	**+**	**+**	**-**	**-**	**-**		ndhC	**+**	**+**	**+**	**+**	**+**	**+**	**+**	**+**	**+**	**+**
	**trnKUUUi37**	**+**	**+**	**+**	**+**	**+**	**+**	**+**	**-**	**-**	**-**		ndhD	**+**	**+**	**+**	**+**	**+**	**+**	**+**	**+**	**+**	**+**
	trnL-CAA	**+**	**+**	**+**	**+**	**+**	**+**	**+**	**Ψ**	**Ψ**	**Ψ**		ndhE	**+**	**+**	**+**	**+**	**+**	**+**	**+**	**+**	**+**	**+**
	trnL-UAA ^**(b)**^	**+**	**+**	**+**	**+**	**+**	**+**	**+**	**+**	**+**	**+**		ndhF	**+**	**+**	**+**	**+**	**+**	**+**	**+**	**+**	**+**	**+**
	**trnLUAAi35**	**+**	**+**	**+**	**+**	**+**	**+**	**+**	**+**	**+**	**+**		ndhG	**+**	**+**	**+**	**+**	**+**	**+**	**+**	**+**	**+**	**+**
	trnL-UAG	**+**	**+**	**+**	**+**	**+**	**+**	**+**	**+**	**+**	**+**		ndhH	**+**	**+**	**+**	**+**	**+**	**+**	**+**	**+**	**+**	**+**
	trnM-CAU	**+**	**+**	**+**	**+**	**+**	**+**	**+**	**+**	**+**	**+**		ndhI	**+**	**+**	**+**	**+**	**+**	**+**	**+**	**+**	**+**	**+**
	trnN-GUU	**+**	**+**	**+**	**+**	**+**	**+**	**+**	**+**	**+**	**+**		ndhJ	**+**	**+**	**+**	**+**	**+**	**+**	**+**	**+**	**+**	**+**
	trnP-GGG	**+**	**+**	**+**	**+**	**+**	**+**	**+**	**+**	**+**	**+**		ndhK	**+**	**+**	**+**	**+**	**+**	**+**	**+**	**+**	**+**	**+**
	trnP-UGG	**+**	**+**	**+**	**+**	**+**	**+**	**+**	**+**	**+**	**+**	**Ribosomal**	rpl2	**+**	**+**	**+**	**+**	**+**	**+**	**+**	**+**	**+**	**+**
	trnQ-UUG	**+**	**+**	**+**	**+**	**+**	**+**	**+**	**+**	**+**	**+**	**proteins**	**rpl2i397**	**+**	**+**	**+**	**+**	**+**	**+**	**+**	**+**	**+**	**+**
	trnR-ACG	**+**	**+**	**+**	**+**	**+**	**+**	**+**	**+**	**+**	**+**		rpl14	**+**	**+**	**+**	**+**	**+**	**+**	**+**	**+**	**+**	**+**
	trnR-CCG ^**(c, d)**^	**+**	**+**	**+**	**+**	**+**	**+**	**+**	**+**	**+**	**+**		rpl16	**+**	**+**	**+**	**+**	**+**	**+**	**+**	**+**	**+**	**+**
	trnR-UCU	**+**	**+**	**+**	**+**	**+**	**+**	**+**	**+**	**+**	**+**		**rpl16i9**	**+**	**+**	**+**	**+**	**-**	**+**	**+**	**+**	**+**	**+**
	trnS-CGA	**-**	**-**	**+**	**+**	**+**	**+**	**+**	**-**	**-**	**-**		rpl20	**+**	**+**	**+**	**+**	**+**	**+**	**+**	**+**	**+**	**+**
	trnS-GCU	**+**	**+**	**+**	**+**	**+**	**+**	**+**	**+**	**+**	**+**		rpl21	**+**	**+**	**+**	**+**	**+**	**+**	**+**	**+**	**+**	**+**
	trnS-GGA	**+**	**+**	**+**	**+**	**+**	**+**	**+**	**+**	**+**	**+**		rpl22	**+**	**+**	**+**	**+**	**+**	**+**	**+**	**+**	**+**	**+**
	trnS-UGA	**+**	**+**	**+**	**+**	**+**	**+**	**+**	**+**	**+**	**+**		rpl23	**+**	**+**	**+**	**+**	**+**	**+**	**+**	**+**	**+**	**+**
	trnT-GGU	**+**	**-**	**+**	**+**	**+**	**+**	**+**	**+**	**+**	**+**		rpl32	**+**	**+**	**+**	**+**	**+**	**+**	**+**	**+**	**+**	**+**
	trnT-UGU	**+**	**+**	**-**	**+**	**+**	**+**	**+**	**-**	**-**	**-**		rpl33	**+**	**+**	**+**	**+**	**+**	**+**	**+**	**+**	**+**	**+**
	trnV-GAC	**+**	**+**	**+**	**+**	**+**	**+**	**+**	**Ψ**	**-**	**-**		rpl36	**+**	**+**	**+**	**+**	**+**	**+**	**+**	**+**	**+**	**+**
	trnV-UAC	**+**	**+**	**+**	**+**	**+**	**+**	**+**	**+**	**+**	**+**		rps2	**Ψ**	**+**	**+**	**+**	**+**	**+**	**+**	**+**	**+**	**+**
	**trnVUACi37**	**+**	**+**	**+**	**+**	**+**	**+**	**+**	**+**	**+**	**+**		rps3	**+**	**+**	**+**	**+**	**+**	**+**	**+**	**+**	**+**	**+**
	trnW-CCA	**+**	**+**	**+**	**+**	**+**	**+**	**+**	**+**	**+**	**+**		rps4	**+**	**+**	**+**	**+**	**+**	**+**	**+**	**+**	**+**	**+**
	trnY-GUA	**+**	**+**	**+**	**+**	**+**	**+**	**+**	**+**	**+**	**+**		rps7	**+**	**+**	**+**	**+**	**+**	**+**	**+**	**+**	**+**	**+**
**Ribosomal**	rrn4.5	**+**	**+**	**+**	**+**	**+**	**+**	**+**	**+**	**+**	**+**		rps8	**+**	**+**	**+**	**+**	**+**	**+**	**+**	**+**	**+**	**+**
**RNAs**	rrn5	**+**	**+**	**+**	**+**	**+**	**+**	**+**	**+**	**+**	**+**		rps11	**+**	**+**	**+**	**+**	**+**	**+**	**+**	**+**	**+**	**+**
	rrn16	**+**	**+**	**+**	**+**	**+**	**+**	**+**	**+**	**+**	**+**		rps12	**+**	**+**	**+**	**+**	**+**	**+**	**+**	**+**	**+**	**+**
	rrn23	**+**	**+**	**+**	**+**	**+**	**+**	**+**	**+**	**+**	**+**		**rps12i114**^**(e)**^	**t**	**t**	**t**	**t**	**t**	**t**	**t**	**t**	**t**	**t**
**Photosystem I**	psaA	**+**	**+**	**+**	**+**	**+**	**+**	**+**	**+**	**+**	**+**		**rps12i346**	**+**	**+**	**-**	**-**	**-**	**-**	**+**	**+**	**+**	**+**
	psaB	**+**	**+**	**+**	**+**	**+**	**+**	**+**	**+**	**+**	**+**		rps14	**+**	**+**	**+**	**+**	**+**	**+**	**+**	**+**	**+**	**+**
	psaC	**+**	**+**	**+**	**+**	**+**	**+**	**+**	**+**	**+**	**+**		rps15	**+**	**+**	**+**	**+**	**+**	**+**	**+**	**+**	**+**	**+**
	psaI	**+**	**+**	**+**	**+**	**+**	**+**	**+**	**+**	**+**	**+**		rps16	**Ψ**	**+**	**-**	**-**	**-**	**-**	**+**	**+**	**+**	**+**
	psaJ	**+**	**+**	**+**	**+**	**+**	**+**	**+**	**+**	**+**	**+**		**rps16i40**	**Ψ**	**+**	**-**	**-**	**-**	**-**	**+**	**+**	**+**	**+**
	psaM	**+**	**+**	**+**	**+**	**+**	**+**	**+**	**+**	**-**	**-**		rps18	**+**	**+**	**+**	**+**	**+**	**+**	**+**	**+**	**+**	**+**
**Photosystem II**	psbA	**+**	**+**	**+**	**+**	**+**	**+**	**+**	**+**	**+**	**+**		rps19	**+**	**+**	**+**	**+**	**+**	**+**	**+**	**+**	**+**	**+**
	psbB	**+**	**+**	**+**	**+**	**+**	**+**	**+**	**+**	**+**	**+**	**RNA**	rpoA	**+**	**+**	**+**	**+**	**+**	**+**	**+**	**+**	**+**	**+**
	psbC	**+**	**+**	**+**	**+**	**+**	**+**	**+**	**+**	**+**	**+**	**polymerase**	rpoB	**+**	**+**	**+**	**+**	**+**	**+**	**+**	**+**	**+**	**+**
	psbD	**+**	**+**	**+**	**+**	**+**	**+**	**+**	**+**	**+**	**+**		rpoC1	**+**	**+**	**+**	**+**	**+**	**+**	**+**	**+**	**+**	**+**
	psbE	**+**	**+**	**+**	**+**	**+**	**+**	**+**	**+**	**+**	**+**		**rpoC1i432**	**+**	**+**	**+**	**+**	**+**	**+**	**+**	**+**	**+**	**+**
	psbF	**+**	**+**	**+**	**+**	**+**	**+**	**+**	**+**	**+**	**+**		rpoC2	**+**	**+**	**+**	**+**	**+**	**+**	**+**	**+**	**+**	**+**
	psbH	**+**	**+**	**+**	**+**	**+**	**+**	**+**	**+**	**+**	**+**	**Miscellaneous**	infA	**Ψ**	**+**	**+**	**+**	**+**	**+**	**+**	**+**	**+**	**+**
	psbI	**+**	**+**	**+**	**+**	**+**	**+**	**+**	**+**	**+**	**+**	**proteins**	ccsA	**+**	**+**	**+**	**+**	**+**	**+**	**+**	**+**	**+**	**+**
	psbJ	**+**	**+**	**+**	**+**	**+**	**+**	**+**	**+**	**+**	**+**		matK	**+**	**+**	**+**	**+**	**+**	**+**	**+**	**+**	**+**	**+**
	psbK	**+**	**+**	**+**	**+**	**+**	**+**	**+**	**+**	**+**	**+**		clpP	**+**	**+**	**+**	**+**	**+**	**+**	**+**	**+**	**+**	**+**
	psbL	**+**	**+**	**+**	**+**	**+**	**+**	**+**	**+**	**+**	**+**		**clpPi71**	**+**	**+**	**+**	**+**	**+**	**+**	**+**	**+**	**+**	**+**
	psbM	**+**	**+**	**+**	**+**	**+**	**+**	**+**	**+**	**+**	**+**		**cplPi363**	**+**	**+**	**+**	**+**	**-**	**-**	**+**	**+**	**+**	**+**
	psbN	**+**	**+**	**+**	**+**	**+**	**+**	**+**	**+**	**+**	**+**		accD	**Ψ**	**+**	**+**	**+**	**+**	**+**	**+**	**+**	**+**	**+**
	psbT	**+**	**+**	**+**	**+**	**+**	**+**	**+**	**+**	**+**	**+**		cemA	**+**	**+**	**+**	**+**	**+**	**+**	**+**	**+**	**+**	**+**
	psbZ	**+**	**+**	**+**	**+**	**+**	**+**	**+**	**+**	**+**	**+**	**Hypothetical**	ycf1 ^(f)^	**+**	**+**	**+**	**+**	**+**	**+**	**Ψ**	**+**	**+**	**+**
**Cytochrome**	petA	**+**	**+**	**+**	**+**	**+**	**+**	**+**	**+**	**+**	**+**	**proteins**	ycf2	**+**	**+**	**+**	**+**	**+**	**+**	**+**	**+**	**+**	**+**
	petB	**+**	**+**	**+**	**+**	**+**	**+**	**+**	**+**	**+**	**+**		ycf3	**+**	**+**	**+**	**+**	**+**	**+**	**+**	**+**	**+**	**+**
	**petBi6**	**+**	**+**	**+**	**+**	**+**	**+**	**+**	**+**	**+**	**+**		**ycf3i124**	**+**	**+**	**+**	**+**	**+**	**+**	**+**	**+**	**+**	**+**
	petD	**+**	**+**	**+**	**+**	**+**	**+**	**+**	**+**	**+**	**+**		**ycf3i354**	**+**	**+**	**+**	**+**	**+**	**+**	**+**	**+**	**+**	**+**
	**petDi8**	**+**	**+**	**+**	**+**	**+**	**+**	**+**	**+**	**+**	**+**		ycf4	**+**	**+**	**+**	**+**	**+**	**+**	**+**	**+**	**+**	**+**
	petG	**+**	**+**	**+**	**+**	**+**	**+**	**+**	**+**	**+**	**+**		ycf12	**+**	**+**	**+**	**+**	**+**	**+**	**+**	**+**	**+**	**+**
	petL	**+**	**+**	**+**	**+**	**+**	**+**	**+**	**+**	**+**	**+**		ycf66	**+**	**+**	**-**	**-**	**Ψ**	**Ψ**	**+**	**Ψ**	**-**	**-**
	petN	**+**	**+**	**+**	**+**	**+**	**+**	**+**	**+**	**+**	**+**		**ycf66i106**	**+**	**+**	**-**	**-**	**Ψ**	**?**	**+**	**Ψ**	**-**	**-**
**Rubisco**	rbcL	**+**	**+**	**+**	**+**	**+**	**+**	**+**	**+**	**+**	**+**												

a) Species: *Isoetes flaccida* (If), *Huperzia lucidula* (Hl), *Ophioglossum californicum* (Oc), *Psilotum nudum* (Pn), *Equisetum hyemale* (Eh), *Equisetum arvense* (Ea), *Angiopteris evecta* (Ae), *Alsophila spinulosa* (As), *Adiantum capillus-veneris* (Ac) and *Pteridium aquilinum* (Pa).

b) CAA anticodon of *trnL*-UAA in *Adiantum capillus-veneris* (Ac) is subjected to partial C-to-U RNA editing [35] and is potentially edited in *Alsophila spinulosa* (As).

c) anticodon of *trnR*-CCG in *Isoetes flaccida* (If) is assumed to be subjected to U-to-C RNA editing [18].

d) Mutations in anticodon of *trnR*-CCG created a UCG anticodon in *Alsophila spinulosa* (As) and *Pteridium aquilinum* (Pa) and a UCA anticodon in *Adiantum capillus-veneris* (Ac).

e) rps12i114 intron is *trans*-spliced (t).

f) *ycf1* in *Angiopteris evecta* (Ae) may retain functionality as a split gene with two protein products [18].

For plastid-encoded RNAs, all four rRNA genes (*rrn4.5, rrn5, rrn16* and *rrn23*) are duplicated within the IR regions, whereas tRNA content varies among monilophytes for five genes (Table 2). The *trnT*-UGU gene was lost from *Ophioglossum* and all completely sequenced Polypodiopsida. The remaining tRNA variation has occurred within Polypodiopsida. This includes the loss of *trnK*-UUU (but not the intron-encoded *matK*) after the divergence of Osmundales [34], the loss of *trnS*-CGA, the fragmentation of *trnL*-CAA which is still intact in *Gleichenia* (HM021798), and the fragmentation and subsequent loss of *trnV*-GAC (Table 2; Figure 2).

The *trnR*-CCG, while present in all leptosporangiate ferns, has undergone several sequential anticodon changes in this group (Additional File 1: Figure S1). The first mutation created a UCG anticodon sequence that is seen in *A. spinulosa* and *P. aquilinum*, which might be corrected by tRNA editing or tolerated by wobble-base pairing. In *A. capillus-veneris* and *Cheilanthes lindheimeri*, a second mutation changed the anticodon into UCA, which would be expected to match UGA stop codons. It is possible that this tRNA is a recent pseudogene [35,36], which is also supported by two mis-pairings in the pseudouridine loop. However, because the *Adiantum* gene is still expressed, Wolf and colleagues suggested it is a functional *trnSeC-*UCA that allows read-through of premature UGA stop codons by inserting selenocysteine [35,36]. Alternatively, we suggest this tRNA still carries arginine as it did ancestrally, only now it recognizes internal UGA stop codons. Thus, this putative *trnR*-UCA may act as a novel failsafe mechanism to ensure arginine is correctly inserted into the protein at any internal UGA codons that were not properly converted by U-to-C RNA editing into CGA (which also codes for arginine). Different mutations have occurred in the anticodon of this tRNA for several other Polypodiales. More work is needed to understand the functional significance of these anticodon shifts.

The set of protein-coding genes in the plastid genome differs for only seven genes among the examined monilophytes (Table 2). The three chlorophyll biosynthesis genes (*chlB, chlL, chlN*) were lost from the cpDNA of *P. nudum.* These genes were also lost from angiosperm plastid genomes in parallel [37] but not from any of the other completely sequenced monilophyte cpDNAs. The *psaM* gene was lost from the sequenced polypods, including *Adiantum*, *Pteridium*, and *Cheilanthes lindheimeri*. The *ycf1* gene in *A. evecta* contains a frameshift mutation that may render it nonfunctional, or it may retain functionality as a split gene with two protein products [18]. Contrary to the conserved presence of most genes, the *ycf66* gene is highly unstable among monilophytes. This gene is intact and likely functional in *A. evecta* and the two lycophytes. However, it is a fragmented pseudogene in Equisetales and *A. spinosa* and it was completely lost from *Ophioglossum*, *Psilotum*, *Adiantum*, and *Pteridium*. A more in-depth study showed that *Botrychium strictum* (another ophioglossoid fern) and several other leptosporangiate ferns have retained an intact gene, indicating that *ycf66* has been independently lost at least four times in monilophyte evolution [38]. The *rpl16* gene also shows a sporadic distribution. It is a pseudogene in the lycophyte *I. flaccida* and completely absent from several fern lineages, including *P. nudum, O. californicum, E. hyemale* and *E. arvense*.

**Figure 4:**
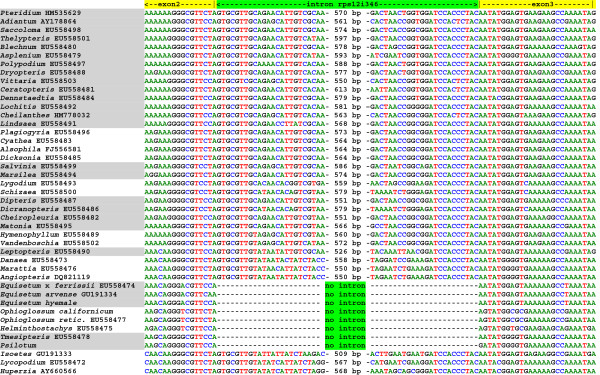
**Distribution of intron rps12i346 in monilophytes.** All available lycophyte and monilophyte plastid *rps12* genes were aligned, and excerpts of the alignment covering the rps12i346 intron sequences and adjacent *rps12* exons are shown. Numbers display the total size of the intron if present in the respective taxon.

The plastome intron content varies for six introns among monilophytes (Table 2). In this study, we use the Dombrovska–Qiu intron nomenclature [39], which names introns based on their nucleotide position within a reference gene (usually from *Marchantia polymorpha*). This nomenclature provides a unified framework to facilitate discussion of orthologous introns, especially when intron content is variable among species as seen here in ferns. The trnK-UUUi37, rps16i40, and ycf66i106 introns were lost from several species due to the loss of the genes that contained them. Like rps16i40, the rps12i346 intron is also absent from *Psilotum, Ophioglossum,* and Equisetales, although in this case the *trans*-spliced *rps12* gene was retained. This shared loss was verified by comparing *rps12* sequences covering this intron region from 40 representative taxa of every major monilophyte group (Figure 4). The intron was found to be absent from the *rps12* gene of all species belonging to Psilotopsida and Equisetopsida, whereas it is still present in all species from Marattiopsida and Polypodiopsida. Finally, both Equisetales cpDNAs have lost the second *clpP* intron (clpPi363), while the loss of rpl16i9 is specific to the newly sequenced *E. hyemale* genome.

### Molecular phylogenetic analyses with additional taxa remain inconclusive regarding monilophyte relationships

**Figure 5:**
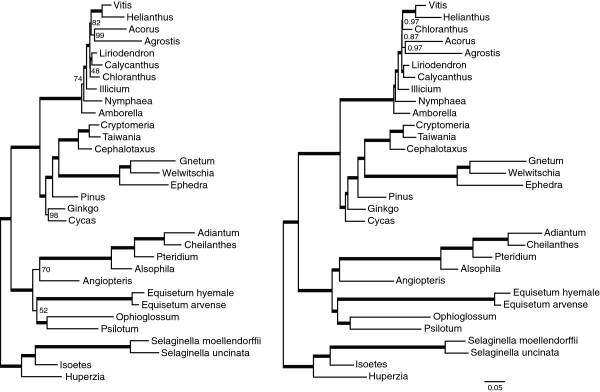
**Phylogenetic analysis of monilophyte plastid genes.** The trees shown were generated by maximum likelihood (left) or Bayesian (right) inference of a data set containing 49 plastid protein genes from 32 vascular plants. Thick branches represent clades with 100% bootstrap support or >0.99 posterior probability. Lower support values are indicated near each node. Trees were rooted on lycophytes. Both trees were drawn to the same scale shown at bottom right.

Phylogenetic analyses were performed using maximum likelihood (ML) with a GTR+G model in RAxML and Bayesian inference (BI) with a CAT-GTR+G model in PhyloBayes (Figure 5). We used the CAT-GTR+G model for Bayesian analyses because it was recently shown to be less susceptible to artifacts caused by long-branch attraction and substitutional saturation [40,41]. At the broadest level, the results were congruent with previous estimates of relationships for the major groups of vascular plants [15,16,18,20,21], including the monophyly of angiosperms, gymnosperms, and ferns *sensu lato* (monilophytes). Among ferns, our analyses grouped *Ophioglossum* and *Psilotum* with strong posterior probability (PP=1.0) and bootstrap support (BS=100) to form a monophyletic Psilotopsida clade, as previously indicated based on analyses of several genes [16,21,22] and large-scale plastome analyses [3,18,25]. In addition, the two *Equisetum* species form a clear monophyletic group (PP=1.0, BS=100), as do the four Polypodiopsida species (PP=1.0, BS=100). Most importantly, both analyses provide evidence (albeit weakly in the ML results) for a sister relationship between Equisetales and Psilotopsida (BS=52, PP=0.99) and between Marattiales and Polypodiopsida (BS=70, PP=1.0), a result that was also recovered in other recent phylogenetic analyses of plastid genes [3,18].

To examine the robustness of these findings, we performed additional RAxML and PhyloBayes analyses on four modified data sets: 1) first and second positions only, 2) third positions only, 3) a reduced sampling of 18 taxa after removal of several fast-evolving seed plants and lycophytes, and 4) translated amino acid sequences for the reduced data set (Additional File 1: Figure S2). Several of these additional RAxML and PhyloBayes analyses corroborated a sister relationship between *Equisetum* and Psilotopsida, while others instead suggested that *Equisetum* is sister to Polypodiopsida, although few results were strongly supported (Table 3). We also reevaluated all five data sets using MrBayes with a GTR+G nucleotide model or CpRev+G amino acid model (Table 3; Additional File 1: Figure S2). The MrBayes results directly parallel the ML results, but with stronger support (PP>0.95) for *Equisetum* + Psilotopsida using the full nucleotide data set and for Equisetum + Polypodiopsida using the first and second or AA data sets. In contrast, the PhyloBayes results with the more advanced CAT-GTR+G model do not provide strong support for *Equisetum* with Polypodiopsida in any analysis.

**Table 3:** **Statistical support for the phylogenetic position of *Equisetum *among ferns**

	**RAxML**	**PhyloBayes**	**MrBayes**
**Data set**	**GTR+G/LG+G**	**CAT-GTR+G**	**GTR+G/CpRev+G**
Nt: All Positions	Equisetum + Psilotopsida	Equisetum + Psilotopsida	Equisetum + Psilotopsida
	BS=52	PP=0.99	PP=0.97
Nt: 1st+2nd Position	Equisetum + Polypodiopsida	Equisetum + Psilotopsida	Equisetum + Polypodiopsida
	BS=58	PP=0.68	PP=0.99
Nt: 3rd Position	Equisetum + Psilotopsida	Equisetum + Psilotopsida	Equisetum + Psilotopsida
	BS=32	PP=0.61	BS=0.49
Nt: Reduced	Equisetum + Psilotopsida	Equisetum + Psilotopsida	Equisetum + Psilotopsida
	BS=44	PP=0.99	PP=0.68
AA: Reduced	Equisetum + Polypodiopsida	Equisetum + Polypodiopsida	Equisetum + Polypodiopsida
	BS=80	PP=0.65	PP=1.0

In summary, it is clear that the relationship among ferns is highly dependent upon choice of model and data when using plastid sequences. The main incongruence among the molecular phylogenetic analyses presented here and previously centers on the enigmatic placement of *Equisetum*. The difficulty in resolving *Equisetum*’s relationship within ferns is likely due to lineage-specific rate heterogeneity and substitutional saturation resulting from a combination of an accelerated substitution rate and a lack of close relatives to *Equisetum*, factors which can lead to phylogenetic inconsistency due to long-branch attraction artifacts.

### Genomic structural changes help resolve relationships among major monilophyte groups

**Figure 6:**
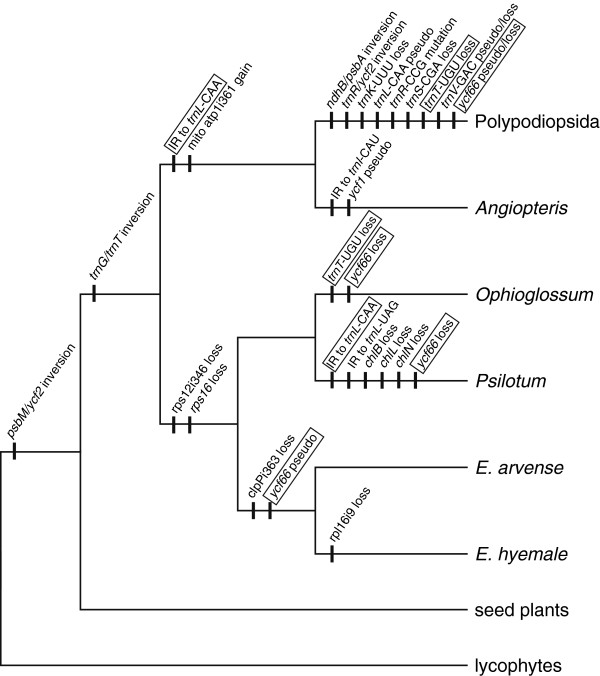
**Phylogenetic history of genomic changes during monilophyte evolution.** The most parsimonious reconstruction of genomic changes was plotted onto the ML topology from Figure 5. Homoplasious changes are boxed. All genomic changes involve the plastid genome, except for the gain of the mitochondrial atp1i361 intron. Genomic changes listed for Polypodiopsida indicate that they are synapomorphic for the four complete cpDNA sequences (*Alsophila spinulosa*, *Adiantum capillus-veneris*, *Pteridium aquilinum* and *Cheilanthes lindheimeri*), but many of them will not necessarily be synapomorphic for all Polypodiopsida.

Given the inconsistent results among molecular phylogenetic analyses, we assessed whether rare genomic structural changes could provide further insight into fern relationships. Indeed, the phylogenetic distribution of genomic structural changes in ferns (Figure 6) provides additional support for the ML and BI topologies recovered in Figure 5. Most interestingly, several structural changes provide new support that help define the position of horsetails and marattioid ferns within monilophytes. The *rps16* gene and the rps12i346 intron are present in the plastid genomes of many land plants, including *Angiopteris* and all examined leptosporangiate ferns (Table 2; Figure 4), indicating that they were probably present in the fern common ancestor. However, *rps16* and rps12i346 are notably absent from all examined ophioglossoid ferns, whisk ferns, and horsetails (Table 2; Figure 4), which is consistent with a single loss for each sequence if *Equisetum* is sister to Psilotopsida (Figure 6). In contrast, at least two independent losses for each sequence would be required if *Equisetum* is more closely related to any other fern group.

Supporting the position of marattioid ferns with leptosporangiate ferns is a novel intron in the mitochondrial *atp1* gene (atp1i361) that is present in both groups but not in any ophioglossoid ferns, whisk ferns, or horsetails (Figure 6; [23]). This distribution, which was previously confusing, can now be explained by a single gain in the common ancestor of leptosporangiate ferns and marattioid ferns. The IR expansion that captured the 3′-*rps12*, *rps7*, *ndhB*, and *trnL*-CAA genes may also be a synapomorphy for these two groups, but further sampling from early diverging leptosporangiate ferns will be necessary to tease apart the timing of this expansion and the two inversions within this group. A similar IR expansion is also found in the *Psilotum* plastid genome, although this is almost certainly a homoplasious event given its absence in *Ophioglossum* and the strong phylogenetic support for a close relationship between these two taxa in all other studies.

Many of the other changes shown in Figure 6 confirm or even presaged relationships that are well established today, such as two previously reported inversions in the LSC that characterize euphyllophytes and monilophytes [18,31]. Similarly, the multiple inversions and tRNA losses shared by all completely sequenced Polypodiopsida species provide further support for their monophyly, and the loss of clpPi363 appears synapormorphic for the genus *Equisetum* (given that species from the two *Equisetum* subgenera lack this intron).

## Conclusions

We sequenced the plastid genomes of three diverse monilophytes: *Equisetum hyemale* (Equisetales), *Ophioglossum californicum* (Ophioglossales), and *Psilotum nudum* (Psilotales). These new genomes revealed limited change in gene and intron content during monilophyte evolution. The structure of the genome is also extremely conserved in *E. hyemale* and *O. californicum*, whose IR boundaries are nearly identical to those in the lycophyte *H. lucidula* and most non-vascular plants. The stability of the IR boundary strongly suggests the retention of this arrangement from the common ancestor of land plants, vascular plants, and ferns *sensu lato*. In contrast, the IR boundaries in *P. nudum*, *Angiopteris evecta*, and leptosporangiate ferns have undergone several expansions to capture genes ancestrally present in the SSC or LSC.

By expanding taxon sampling to include the first ophioglossoid fern and a second representative from *Equisetum*, we hoped to provide more definitive resolution of taxonomic relationships among the major groups of ferns. While the results of the phylogenetic analyses provided generally weak and inconsistent support for the positions of *Equisetum* and *Angiopteris*, their phylogenetic affinities were revealed by mapping rare genomic structural changes in a phylogenetic context: the presence of a unique mitochondrial *atp1* intron argues strongly for a sister relationship between Polypodiopsida and Marattiopsida, and the absence of the *rps16* gene and the rps12i346 intron from *Equisetum, Psilotum,* and *Ophioglossum* indicates that Equisetopsida is sister to Psilotopsida.

Further plastome sequencing of marattioid ferns and early diverging leptosporangiate ferns will likely be necessary to solidify the sister relationship between these two lineages, but the position of *Equisetum* is unlikely to be resolvable with more plastome data. This is due to unavoidable long-branch artifacts for Equisetopsida caused by the increased plastid sequence diversity in this group and by the lack of any close, living relatives of *Equisetum*. Expanded sequencing from mitochondrial and nuclear genomes may prove to be more useful, although this remains to be tested.

## Methods

### Source of plants

*Ophioglossum californicum* plants and a single *Psilotum nudum* plant were obtained from the living collection at the Beadle Center Greenhouse (University of Nebraska–Lincoln). *Equisetum hyemale* plants were ordered from Bonnie’s Plants (Newton, NC, USA) and grown to maturity in the Beadle Center Greenhouse.

### DNA extraction and sequencing

For each plant, a mixed organelle fraction was prepared by differential centrifugation using buffers and techniques described previously [42,43]. Mature, above-ground tissue (50–100 g) was homogenized in a Waring blender, filtered through four layers of cheesecloth, and then filtered through one layer of Miracloth. The filtrate was centrifuged at 2,500 × g in a Sorvall RC 6+ centrifuge for 15 min to remove nuclei, most plastids, and cellular debris. The supernatant was centrifuged at 12,000 × g for 20 min to pellet mitochondria and remaining plastids.

Organelle-enriched DNA was isolated from the mixed organelle fraction using a simplified version of the hexadecyltrimethylammonium bromide (CTAB) procedure described previously [44]. Briefly, the mixed organelle fraction was placed in isolation buffer for 30 min at 65°C with occasional mixing. The solution was centrifuged for 3 min and the supernatant was treated twice with an equal volume of 24:1 chloroform:isoamyl alcohol. DNA was precipitated with 0.6 volume isopropanol overnight at −20°C, pelleted by centrifugation for 10 min at 10,000 x g, washed twice with 70% ethanol, and then resuspended in DNase-free H_2_O. A quantitative PCR assay [43] using species-specific primers targeting nuclear, mitochondrial, and plastid genes confirmed that the organelle-enriched DNA contained similar copy numbers of mitochondrial and plastid genomes and greatly reduced levels of nuclear genomic DNA (data not shown).

Organelle-enriched DNAs were sequenced using the Illumina platform at the BGI Corporation (for *E. hyemale* and *P. nudum*) or at the University of Illinois Roy J. Carver Biotechnology Center (for *O. californicum*). For each species, ~20 million paired-end sequence reads of 100 bp were generated from sequencing libraries with median insert sizes of 760 bp to 910 bp (Additional File 1: Table S1). In addition, *O. californicum* organelle-enriched DNA was sent to the University of Nebraska Core for Applied Genomics and Ecology for 454 sequencing on the Roche-454 GS FLX platform using Titanium reagents, which produced ~270,000 single-pass reads with average length of 316 bp (Additional File 1: Table S1).

### Genome assembly

The organelle-enriched Illumina sequencing reads from *O.californicum*, *P. nudum*, and *E. hyemale* were assembled with Velvet [45] using a large range of parameters, and the best results were individually chosen. The scaffolding option of Velvet was usually used to combine contigs into larger scaffolds based on the paired-end information of the sequence libraries. Nuclear contamination in the sequence data resulted in scaffolds with low coverage, which were discarded. Remaining scaffolds with high coverage were used for blastn searches against the cpDNA of *P. nudum* (NC_003386) or *E. arvense* (NC_014699) to identify scaffolds containing plastid DNA.

To assemble the *O. californicum* plastid genome, we used Velvet with a kmer length of 57 bp, resulting in a maximum scaffold size of 123,523 bp that spanned most of the LSC and SSC and the entire IR. The IR had double the coverage compared with the remaining scaffold and was used twice in the complete cpDNA sequence. An additional scaffold of 4,684 bp was identified covering the remaining part of the SSC. To finish the genome, all gaps between and within scaffolds were eliminated using a draft assembly of the 454 sequencing data put together by Roche’s GS de novo Assembler v2.3 (“Newbler”) with default parameters.

The cpDNA of *P. nudum* was assembled from five overlapping cpDNA contigs identified in two Velvet assemblies using either a kmer length of 75 bp with scaffolding or a kmer length of 67 bp without scaffolding. The size of the scaffolds varied from 1,687 bp to 84,740 bp. One of these scaffolds with a size of 18,935 bp had twice the coverage and exactly covered the IR region. This scaffold was used twice when all contigs were adjusted according to their overlapping end regions. No further gap filling was necessary to finish the genome.

We used Velvet with a kmer length of 37 bp without scaffolding to assemble the cpDNA of *E. hyemale.* Scaffolding was done by SSPACE [46] since it was able to connect more contigs into larger scaffolds than using Velvet with the scaffolding option. Three scaffolds produced by SSPACE covered most of the plastid genome. These contigs were arranged by aligning them to the *E. arvense* database entry (NC_014699). The first 10,093 bp of one contig covered the IR region and was used twice in the completed sequence. To finish this genome, gaps between or within the three scaffold sequences were closed by polymerase chain reaction (PCR) using GoTaq DNA polymerase according to the manufacturer’s protocol (Promega, Madison, Wisconsin, USA).

To evaluate assembly quality and accuracy, Illumina sequencing reads were mapped onto the three finished cpDNA sequences with Bowtie 2.0.0 [47]. The mapped reads provided an average coverage of 344x, 188x, and 450x for the genomes of *E. hyemale*, *O. californicum*, and *P. nudum*, respectively (Additional File 1: Figure S3). All parts of the genome were covered at roughly equal depth suggesting the finished genomes were assembled accurately and completely. However, there were a few nucleotides where the consensus sequence constructed by velvet and/or SSPACE disagreed with the majority of mapped reads. At these positions, we used the mapped read sequences to correct the consensus genome sequence.

### Genome annotation

The location of *O. californicum* protein-coding, rRNA, and tRNA genes were initially determined using DOGMA annotation software [48]. Existing GenBank entries of complete cpDNAs were used as a template for a preliminary annotation of the complete plastid sequences of *P. nudum* and *E. hyemale* sequenced in this study. For any tRNA gene annotations in these three genomes that conflicted with annotations in previously sequenced ferns, we manually examined their secondary structures and anticodons to assess identity and functionality. Finally, to ensure annotation consistency among the lycophyte and monilophyte cpDNAs compared here, gene and intron presence was individually re-evaluated using blastn and blastx searches. The annotated genomic sequences were deposited in GenBank under accession numbers KC117177 (*E. hyemale*), KC117178 (*O. californicum*), and KC117179 (*P. nudum*).

### Phylogenetic analysis

We downloaded the data set from Karol et al. [18] and made the following modifications: 1) removed all ten bryophyte and green algal species, which are distantly related to ferns, to avoid complications with distant outgroups, 2) removed nine angiosperms from the densely sampled eudicot and monocot lineages to speed up analyses, 3) added four new ferns (*Cheilanthes lindheimeri*, *E. hyemale*, *O. californicum*, *Pteridium aquilinum*) to improve fern sampling, 4) added three new Coniferales (*Cephalotaxus wilsoniana*, *Cryptomeria japonica*, and *Taiwania cryptomeroides*) to improve gymnosperm sampling, 5) added *Calycanthus floridus* to improve magnoliid sampling in angiosperms, 6) replaced the *P. nudum* sequences obtained from an unpublished genome with data from our newly sequenced *P. nudum* plastome, and 7) replaced the *Adiantum* cDNA sequences with genomic DNA sequences to avoid mixing of DNA and cDNA in the phylogenetic analyses. All genes were aligned in Geneious [49] and matrices were concatenated in SequenceMatrix [50]. Aligned sequences were manually adjusted when necessary, and poorly aligned regions were removed using Gblocks [51] in codon mode with relaxed parameters (b2 = half+1, b4 = 5, b5 = half). The final data set contained 49 plastid genes from 32 taxa totaling 32,547 bp. Additional data sets were constructed that included 1^st^ and 2^nd^ codon positions only, 3^rd^ codon positions only, a reduced sampling of 18 taxa after eliminating the fastest evolving seed plants and lycophytes, or an amino acid translation of the reduced data set. GenBank accession numbers for data used in the alignment are provided in (Additional File 1: Table S2), and the data set was deposited in treeBASE (Study ID 13741).

Phylogenetic analyses were performed using maximum likelihood (ML) and Bayesian inference (BI). ML trees were estimated with RAxML [52] using the GTR+G model for nucleotide data sets and the LG+G model for the amino acid data set. For each analysis, 1000 bootstrap replicates were performed using the fast bootstrapping option [53]. BI was performed with PhyloBayes [41] using the GTR-CAT+G4 model for all data sets, which was recently shown to outperform all other models during Bayesian analyses and to be less influenced by long-branch attraction and substitutional saturation artifacts [40,41]. For each data set, two independent chains were run until the maximum discrepancy between bipartitions was <0.1 (minimum 75,000 generations). The first 200 sampled trees were discarded as the burn-in. BI was also performed with MrBayes [54]. For each analysis, two runs with 4 chains were performed in parallel, and the first 25% of all sampled trees were discarded as the burn-in. Nucleotide data sets used the GTR+G model and were run for 500,000 generations with trees sampled every 500 generations. The amino acid data set used the CpRev+G model and was run for 100,000 generations with trees sampled every 100 generations. All ML and BI trees were rooted on lycophytes.

## Competing interests

The authors declare that they have no competing interests.

## Authors’ contributions

FG and JPM designed the study. FG performed most analyses and prepared most figures and tables. WG, AKH, and JPM performed some computational analyses and prepared some figures and tables. EAG performed some experimental analyses. FG, WG, AKH, and JPM analyzed results and contributed to the writing. All authors have read and approved the final version of the manuscript.

## Supplementary Material

Additional file 1 **Table S1.** DNA sequencing information. **Table S2.** Genome sequences used in this study. **Figure S1.** Alignment of plastid *trnR*-CCG in monilophytes. Selected *trnR*-CCG sequences from representative monilophyte taxa were aligned to the sequences from the lycophytes *Huperzia lucidula* and *Isoetes flaccida*. Alignment positions with >70% identity among sequences are shaded in grey. Predicted tRNA secondary structure is depicted in dot-bracket format above and below the alignment. The tRNA anticodon position is indicated by “AAA” and highlighted in yellow. A deletion in the *Cryptogramma* gene is indicated by dashes, whereas two insertion sequences (the first in the top five Polypodiopsida species and the second in *Polybotrya* only) are boxed in red with a red bar indicating their position within the gene sequences. **Figure S2.** Additional phylogenetic analyses. A) Nt - all positions for MrBayes (RAxML and PhyloBayes results shown in Figure 5). B) Nt - 1st and 2nd positions. C) Nt - 3rd positions. D) Nt - reduced taxon sampling. E) AA - reduced taxon sampling. **Figure S3.** Depth of sequencing coverage for fern plastomes. Illumina sequencing reads were mapped onto the finished genomes using Bowtie 2.0.0 [47]. Depth of coverage was estimated using a window size of 100 and a step size of 10; it is reported on a logarithmic base 2 scale. Mean coverage for each genome is indicated by the dashed horizontal line. Genome position is given in kilobases. Click here for file
