# Supplementary material for: Complete plastid genomes from Ophioglossum californicum, Psilotum nudum, and Equisetum hyemale reveal an ancestral land plant genome structure and resolve the position of Equisetales among monilophytes
Source: BMC Evol Biol. 2013 Jan 11;13:8. doi: 10.1186/1471-2148-13-8 (PMC3553075; doi:10.1186/1471-2148-13-8)

Supplementary Table 1. DNA sequencing information.

	Equisetum	*Ophioglossum* 1	*Ophioglossum* 2	*Psilotum*
**Sequencing facility**	BGI Americas (bgiamericas.com)	RJ Carver Biotechnology Center, U. Illinois (www.biotech.uiuc.edu)	Core for Applied Genomics and Ecology, U. Nebraska (cage.unl.edu)	BGI Americas (bgiamericas.com)
**Platform**	Illumina HiSeq2000	Illumina HiSeq2000	454 GS FLX	Illumina HiSeq2000
**Seq. type**	paired-end llumina	paired-end llumina	single pass 454	paired-end llumina
**No. reads**	21.0 M	17.9 M	0.268 M	23.1 M
**Read len.**	100 bp	100 bp	316 bp (mean)	100 bp
**Total data**	2100 M	1790 M	84.3 M	2310 M
**Library size**	762 bp (median)	910 bp (median)	N/A	760 bp (median)

Supplementary Table 2. Genome sequences used in this study.

Species	GenBank Acc No	Usage
**Angiosperms**		
Acorus americanus	DQ069409	Figure 4
Agrostis stolonifera	EF115543	Figure 4
Amborella trichopoda	AJ506156	Figure 4
Calycanthus floridus	AJ428413	Figure 4
Chloranthus spicatus	EF380352	Figure 4
Helianthus annuus	DQ383815	Figure 4
Illicium oligandrum	EF380354	Figure 4
Liriodendron tulipifera	DQ899947	Figure 4
Nymphaea alba	AJ627251	Figure 4
Vitis vinifera	DQ424856	Figure 4
**Gymnosperms**		
Cephalotaxus wilsoniana	AP012265	Figure 4
Cryptomeria japonica	AP009377	Figure 4
Cycas taitungensis	AP009339	Figures 3 and 4
Ephedra equisetina	AP010819	Figure 4
Ginkgo biloba	AB684440	Figure 4
Gnetum parvifolium	AP009569	Figure 4
Pinus thunbergii	D17510	Figure 4
Taiwania cryptomerioides	AP012266	Figure 4
Welwitschia mirabilis	EU342371	Figure 4
**Ferns**		
Adiantum capillus-veneris	AY178864	Figure 4
Alsophila spinulosa	FJ556581	Figure 4
Angiopteris evecta	DQ821119	Figures 3 and 4
Cheilanthes lindheimeri	HM778032	Figure 4
Equisetum arvense	GU191334	Figures 3 and 4
Equisetum hyemale	KC117177	Figures 3 and 4
Ophioglossum californicum	KC117178	Figures 3 and 4
Psilotum nudum	KC117179	Figures 3 and 4
Pteridium aquilinum	HM535629	Figure 4
**Lycophytes**		
Huperzia lucidula	AY660566	Figures 3 and 4
Isoetes flaccida	GU191333	Figure 4
Selaginella moellendorffii	FJ755183	Figure 4
Selaginella uncinata	AB197035	Figure 4
**Hornworts**		
Anthoceros formosae	AB086179	Figure 3
**Mosses**		
Physcomitrella patens	AP005672	Figure 3
Syntrichia ruralis	FJ546412	Figure 3
**Liverworts**		
Aneura mirabilis	EU043314	Figure 3
Marchantia polymorpha	X04465	Figure 3
Ptilidium pulcherrimum	HM222519	Figure 3

Supplementary Figure 1 – Alignment of plastid *trnR*-CCG in monilophytes. Selected *trnR*-CCG sequences from representative monilophyte taxa were aligned to the sequences from the lycophytes *Huperzia lucidula* and *Isoetes flaccida*. Alignment positions with >70% identity among sequences are shaded in grey. Predicted tRNA secondary structure is depicted in dot-bracket format above and below the alignment. The tRNA anticodon position is indicated by “AAA” and highlighted in yellow. A deletion in the *Cryptogramma* gene is indicated by dashes, whereas two insertion sequences (the first in the top five Polypodiopsida species and the second in *Polybotrya* only) are boxed in red with a red bar indicating their position within the gene sequences.

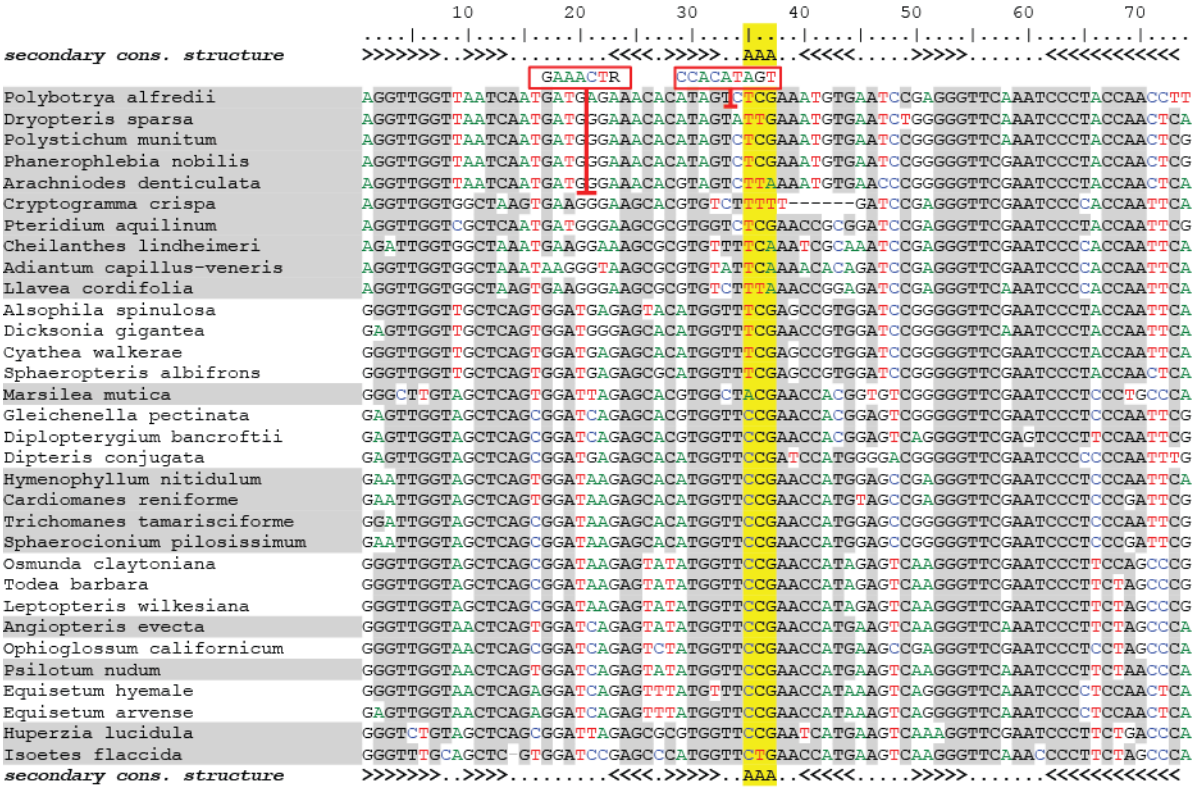

Supplementary Figure 2 – Additional phylogenetic analyses. A) Nt - all positions for MrBayes (RAxML and PhyloBayes results shown in Figure 5). B) Nt - 1st and 2nd positions. C) Nt - 3rd positions. D) Nt - reduced taxon sampling. E) AA - reduced taxon sampling.

A Nt - All Positions

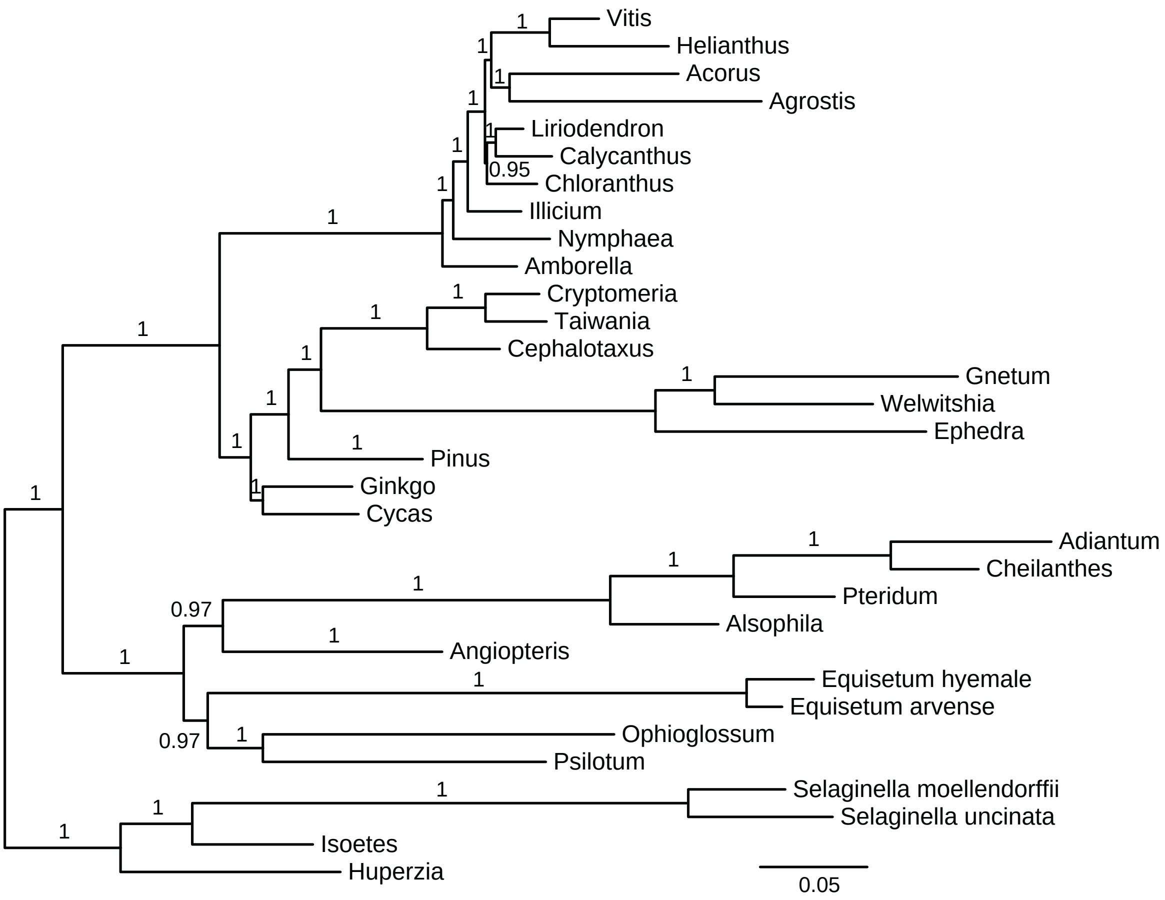

MrBayes (GTR+G)

B Nt - 1st + 2nd Position

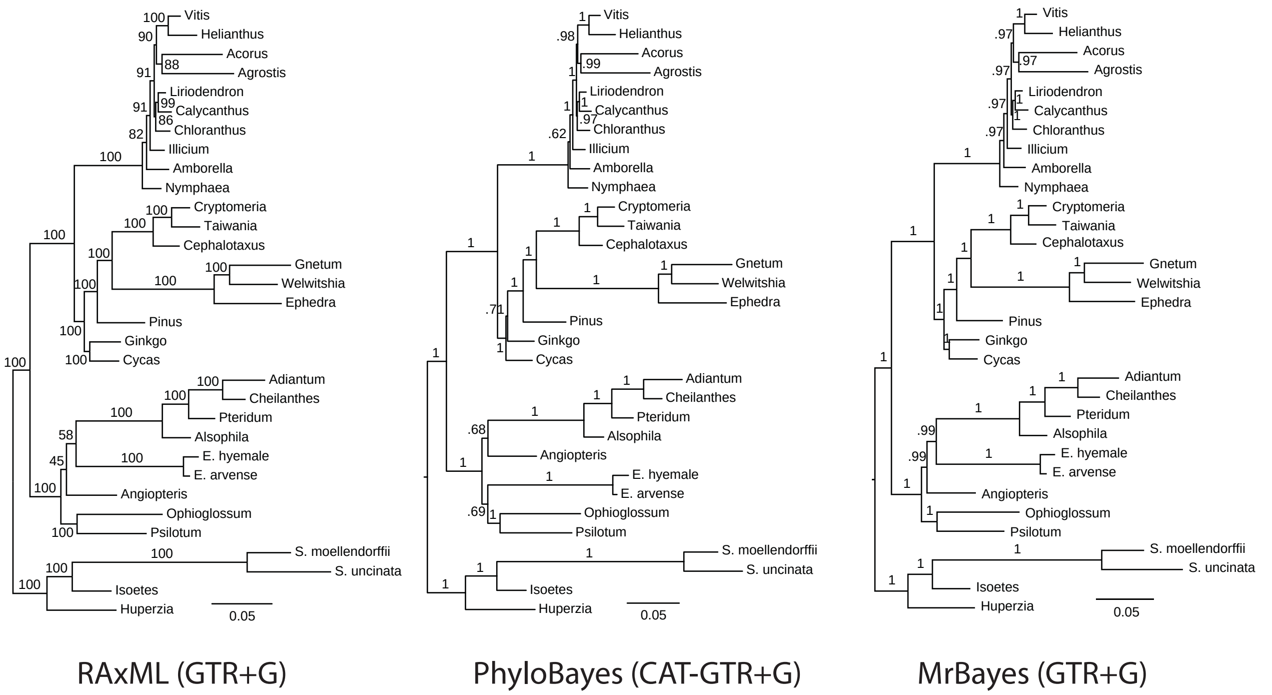

C Nt - 3rd Position

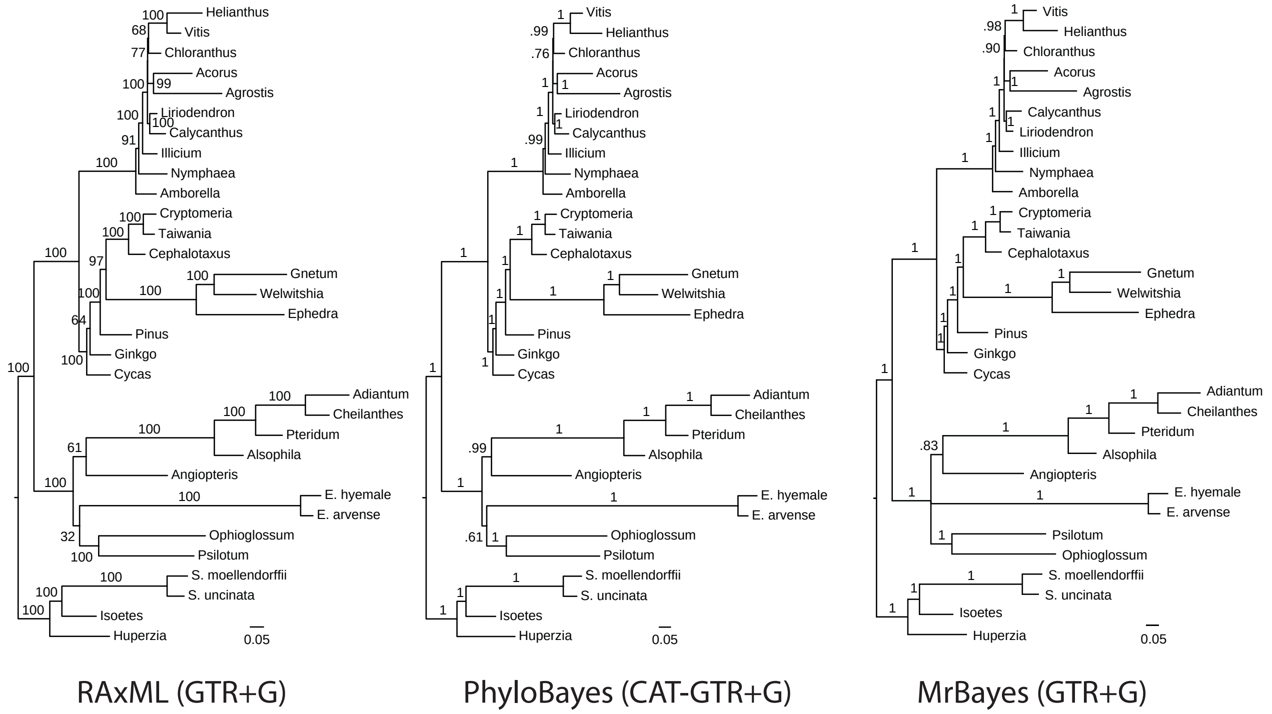

D Nt - Reduced

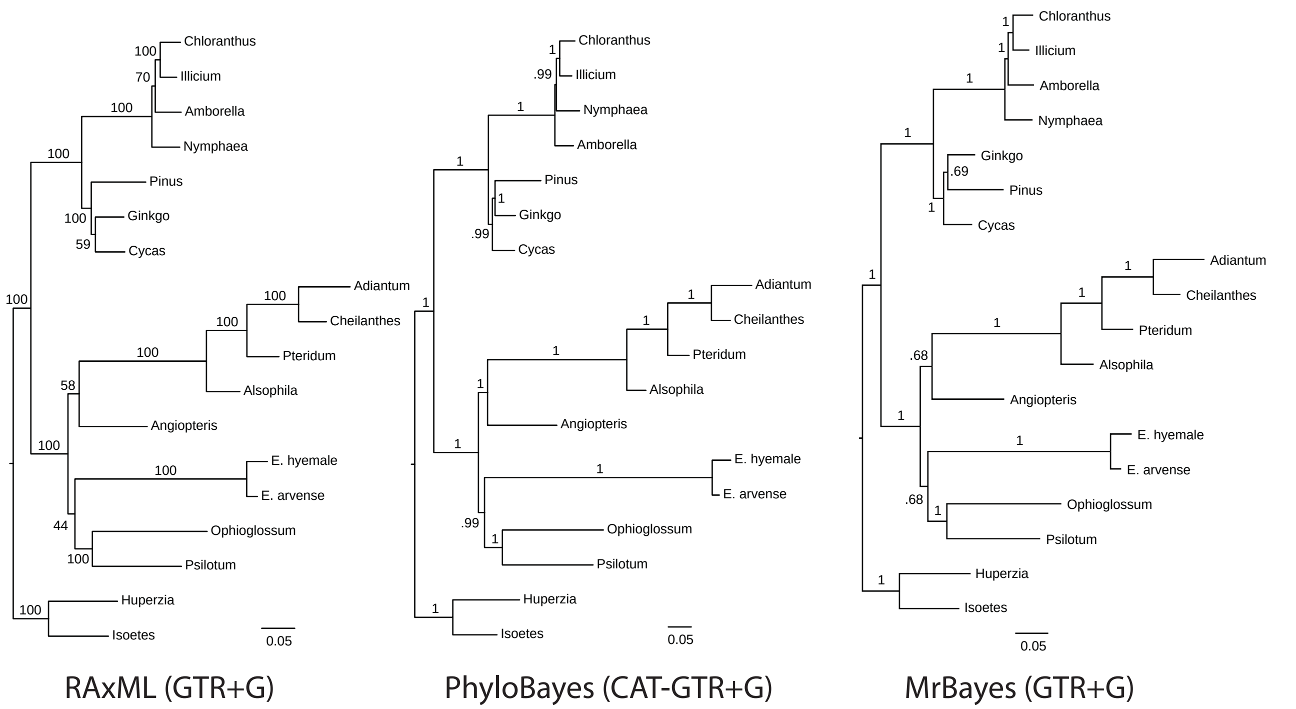

E AA - Reduced

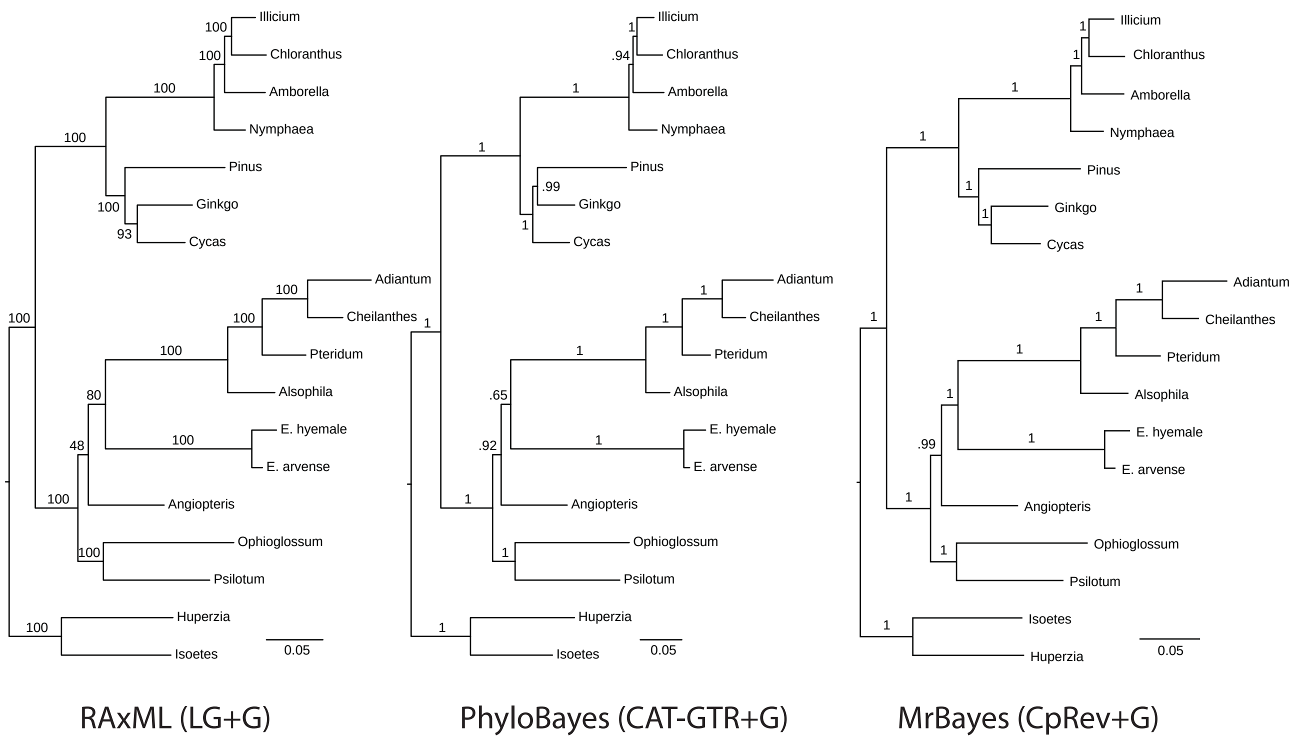

Supplementary Figure 3 – Depth of sequencing coverage for fern plastomes. Illumina sequencing reads were mapped onto the finished genomes using Bowtie 2.0.0 (Langmead et al 2012). Depth of coverage was estimated using a window size of 100 and a step size of 10; it is reported on a logarithmic base 2 scale. Mean coverage for each genome is indicated by the dashed horizontal line. Genome position is given in kilobases.

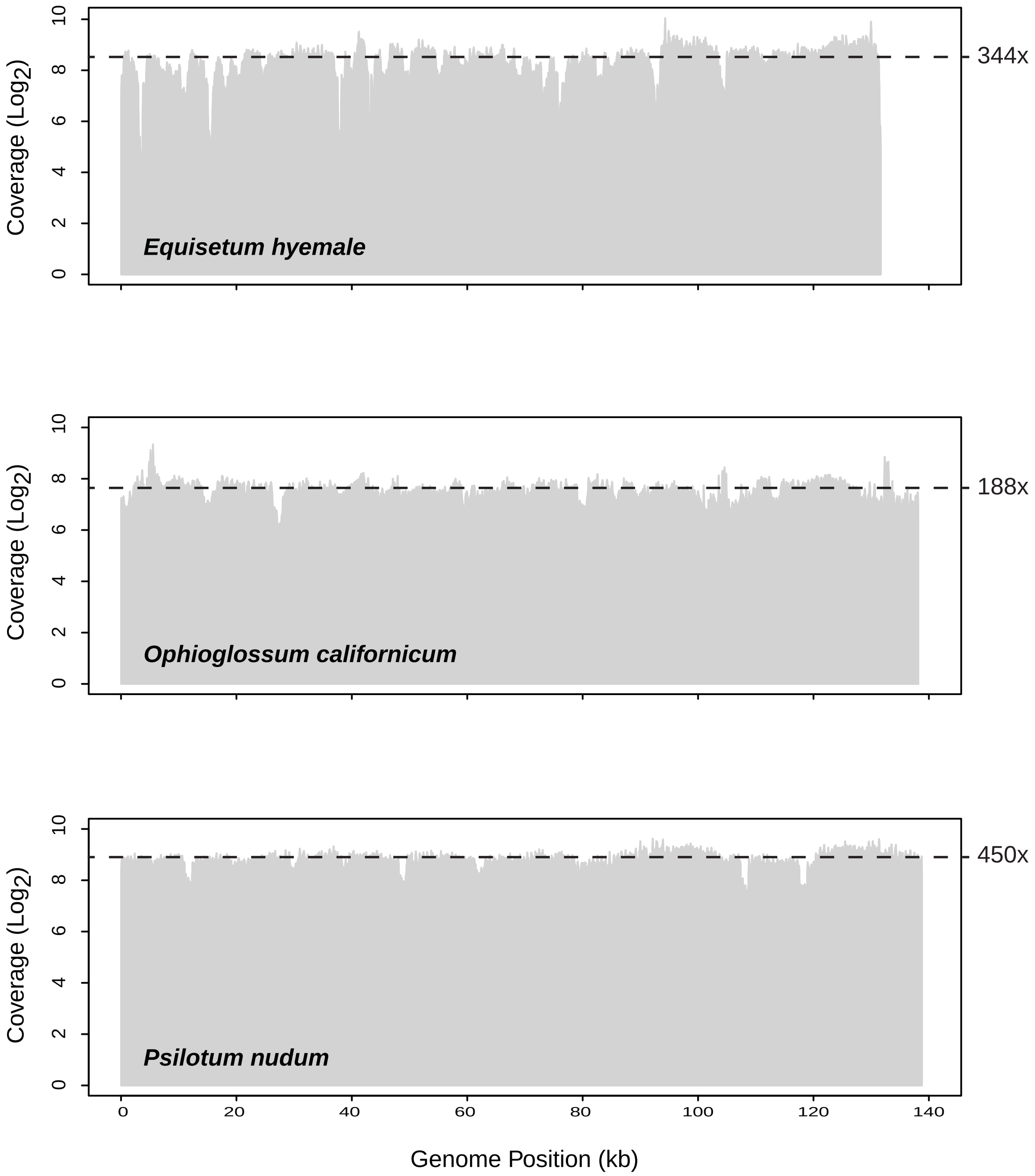

Supplement: Additional file 1 — Table S1. DNA sequencing information. Table S2. Genome sequences used in this study. Figure S1. Alignment of plastid trnR-CCG in monilophytes. Selected trnR-CCG sequences from representative monilophyte taxa were aligned to the sequences from the lycophytes Huperzia lucidula and Isoetes flaccida. Alignment positions with >70% identity among sequences are shaded in grey. Predicted tRNA secondary structure is depicted in dot-bracket format above and below the alignment. The tRNA anticodon position is indicated by “AAA” and highlighted in yellow. A deletion in the Cryptogramma gene is indicated by dashes, whereas two insertion sequences (the first in the top five Polypodiopsida species and the second in Polybotrya only) are boxed in red with a red bar indicating their position within the gene sequences. Figure S2. Additional phylogenetic analyses. A) Nt - all positions for MrBayes (RAxML and PhyloBayes results shown in Figure 5). B) Nt - 1st and 2nd positions. C) Nt - 3rd positions. D) Nt - reduced taxon sampling. E) AA - reduced taxon sampling. Figure S3. Depth of sequencing coverage for fern plastomes. Illumina sequencing reads were mapped onto the finished genomes using Bowtie 2.0.0 [47]. Depth of coverage was estimated using a window size of 100 and a step size of 10; it is reported on a logarithmic base 2 scale. Mean coverage for each genome is indicated by the dashed horizontal line. Genome position is given in kilobases. [file 1471-2148-13-8-S1.pdf]
